# Diagnostic Accuracy of Point of Care Cryptococcal Antigen Lateral Flow Assay in Fingerprick Whole Blood and Urine Samples for the Detection of Asymptomatic Cryptococcal Disease in Patients with Advanced HIV Disease

**DOI:** 10.1128/spectrum.01075-22

**Published:** 2022-08-04

**Authors:** Kathryn Boyd, Vinie Kouamou, Admire Hlupeni, Zorodzai Tangwena, Chiratidzo E. Ndhlovu, Azure T. Makadzange, CryptoART Study Team

**Affiliations:** a Charles River Medical Group, Harare, Zimbabwe; b Unit of Internal Medicine, Department of Primary Health Care Sciences, University of Zimbabwe, Harare, Zimbabwe; Keck School of Medicine of the University of Southern California

**Keywords:** cryptococcal disease, cryptococcal antigen, advanced HIV disease, point of care, diagnostic accuracy, Zimbabwe

## Abstract

Cryptococcal disease (CD) is a leading cause of mortality among individuals with advanced HIV disease (AHD). Screening with serum cryptococcal antigen (sCrAg) lateral flow assay (LFA) enables early detection of subclinical disease but requires venipuncture and laboratory processing. Clinic-based point of care (POC) CrAg screening tests using urine or fingerprick whole blood could facilitate early diagnosis of CD. We evaluated the diagnostic performance of POC clinic-based fingerprick whole blood and urine CrAg compared to the gold standard laboratory sCrAg LFA in screening for CD among asymptomatic individuals with CD4 counts of <200 cells/μL in Harare, Zimbabwe. sCrAg positive participants who consented to a lumbar puncture also had cerebrospinal fluid (CSF) CrAg testing and titers for CSF-positive specimens. A total of 1,333 individuals were screened, and over half (56.6%) were males. The median (interquartile range) CD4 count was 27.5 (11–46) cells/μL. We found a sensitivity of 63.8% (95% CI: 54.8–72.1) and specificity of 84.0% (95% CI: 81.7–86.0) for urine CrAg, and a sensitivity of 48.0% (95% CI: 39.1–57.1) and specificity of 99.5% (95% CI: 98.9–99.8) was found for fingerprick whole blood. The sensitivity of both POC CrAg tests increased in individuals with sCrAg titers of ≥1:160, CD4 count of <50 cells/μL and disseminated central nervous system (CNS) disease. Clinic-based POC urine and fingerprick whole blood CrAg testing performed better in screening for CD among AHD patients with CNS disease. More sensitive assays to identify AHD patients with asymptomatic CD are needed.

**IMPORTANCE** Cryptococcal disease (CD) remains a leading cause of morbidity and mortality among individuals with advanced HIV disease (AHD). Identifying point of care (POC) approaches to screening for CD in asymptomatic individuals is important to guide therapeutic management. We evaluated the use of POC fingerprick whole blood and urine testing for cryptococcal disease in patients with AHD as compared with laboratory-based serum antigen testing. POC fingerprick whole blood and urine testing had low sensitivity and specificity in asymptomatic individuals with AHD. Most analysis has focused on evaluating test performance in symptomatic individuals. Here we show that POC testing with whole blood and urine samples should not be used to screen for asymptomatic CD in AHD.

## INTRODUCTION

A significant proportion of people living with HIV in low- and middle-income countries (LMICs) continue to be diagnosed with advanced HIV disease (AHD), defined as CD4 of <200 cells/μL or the World Health Organization (WHO) clinical stage 3 or 4 disease for adults initiating antiretroviral therapy (ART) (1). The Zimbabwe Population-Based HIV Impact Assessment Survey (2015–2016) found that 17% of ART-naive patients had AHD (2). In 2020, the estimates from the Zimbabwe national electronic patient monitoring system found that about a third of ART-naive patients were presenting with AHD. WHO estimates that up to 50% of people presenting for ART initiation in sub-Saharan Africa (SSA) have AHD and that these individuals are at increased risk of mortality following ART initiation (3). WHO recommends that people with AHD should be given priority for assessment and ART initiation and in 2017 released guidelines for the management of individuals with AHD (3). These guidelines recommend providing a comprehensive package of care that includes (i) rapid ART initiation with same-day ART initiation for those that are ready, (ii) diagnostic evaluation for tuberculosis (TB) and cryptococcal disease (CD), (iii) prophylaxis and preemptive treatment for TB and CD, and (iv) adherence support. Data from recent trials suggest that a greater proportion (over 60%) of individuals with HIV-associated CD are ART experienced, these ART-experienced but AHD individuals are an increasingly important population as access to ART becomes universal, and ART failure or nonadherence is implicated (4–7).

Diagnostic evaluation and appropriate preemptive treatment for TB and CD are critical in supporting good clinical outcomes for individuals with AHD. Screening for CD involves venipuncture and laboratory-based screening of serum for the detection of cryptococcal antigen (CrAg) using a rapid lateral flow assay (LFA). Since this procedure is laboratory based and often centralized in many African settings, there may be challenges with transporting samples to the laboratory, processing of the samples due to shortage of laboratory staff, and turnaround time of the results to enable timely management of the patient. As a result, point of care (POC) screening could be essential in these settings for early diagnosis and preemptive treatment in individuals at risk of CD. This can be achieved by using samples that do not need further laboratory processing such as urine and whole blood (e.g., from a fingerprick).

The rapid LFA has demonstrated good accuracy on serum (gold standard) and cerebrospinal fluid (CSF) (8–10). However, data on its diagnostic accuracy in urine and whole blood is still limited. Therefore, we conducted a study to evaluate the diagnostic performance of clinic-based CrAg LFA in fingerprick whole blood and urine samples among individuals with CD4 counts below 200 cells/μL in Zimbabwe who had no symptoms suggestive of meningitis. Individuals with CD4 counts below 200 cells/μL were evaluated as they are at highest risk of CD.

## RESULTS

### Demographic and clinical characteristics.

A total of 1,333 individuals were included in this study; 132 (9.9%) were sCrAg positive, with a median sCrAg titer of 1:20 (IQR: 1:5–1:160). Above half (56.6%) of the participants were males. The median (IQR) age of participants at enrollment was 37 (32-43) years and the majority (89%) were ART naive. The median (IQR) CD4 count was 27.5 (11–46) cells/μL (Table 1). Sixty-six sCrAg positive participants consented to a lumbar puncture (LP), and of these, 12 (18.2%) were CSF CrAg positive, with a median CSF titer of 1:240 (IQR: 1:60–1:1920). Their median sCrAg titer was 1:2560 (IQR: 1:240–1:2560) compared to 1:10 (IQR: 1:5–1:40) in those who were CSF CrAg negative, *P* < 0.0001.

**TABLE 1:** Characteristics of the study participants (*n* = 1,333) at enrollment^*a*^

Characteristic	Total	sCrAg positive (*n* = 132)	sCrAg negative (*n* = 1,201)	*P* value
Median (IQR) age in yrs	37 (32–43)	38 (33–43)	37 (31–43)	0.15
Gender Males, n (%)	754 (56.6)	80 (60.6)	674 (56.1%)	0.32
Median (IQR) CD4 count in cells/μL	27.5 (11–46)	27.5 (11–46)	32 (14–57)	0.15

a IQR, interquartile range; sCrAg, serum cryptococcal antigen.

**FIG 1:**
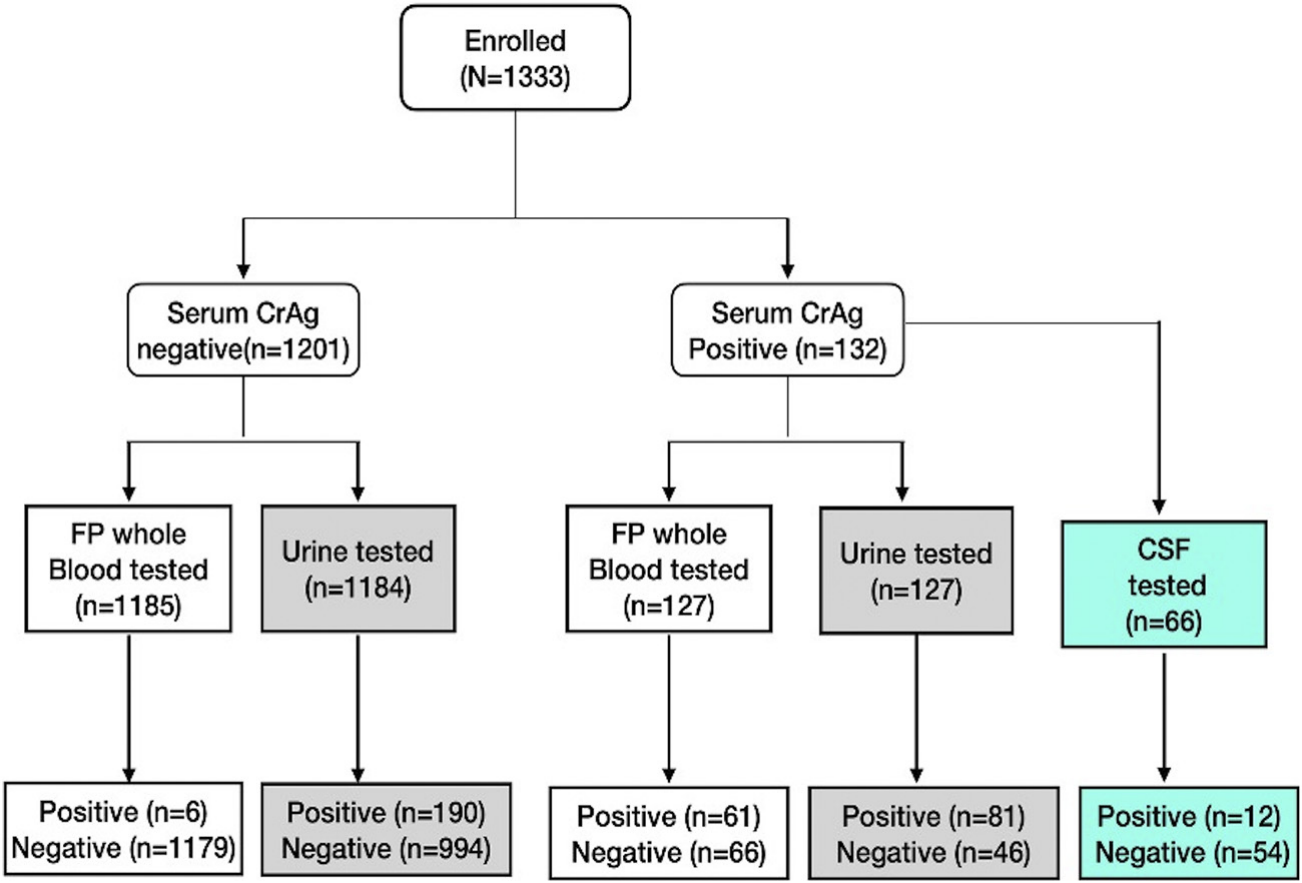
Distribution of the study participants. FP, fingerprick; CrAg, cryptococcal antigen; CSF, cerebrospinal fluid.

Of the 1,201 sCrAg negative participants enrolled, 1,185 and 1,184 had CrAg testing on their fingerprick whole blood and urine samples, respectively. Among the 132 sCrAg positive participants, 127 had their fingerprick whole blood and urine tested for CrAg (11) (Fig. 1).

### Diagnostic performance of fingerprick whole blood and Urine CrAg testing.

**(i) Validity.** ***(a) Urine CrAg testing.*** Of the 127 sCrAg positive samples that underwent urine CrAg testing, 81 (63.8%) tested positive and 46 (36.2%) tested negative (false negative), whereas among the 1,184 sCrAg negative patients, 994 (84%) tested negative and 190 (16%) tested positive (false positive). Overall, urine CrAg testing yielded a sensitivity of 63.8% (95% CI: 54.8–72.1), a specificity of 84.0% (95% CI: 81.7–86.0), a PPV of 29.9% (95% CI: 24.5–35.7), and a NPV of 95.6% (95% CI: 94.1–96.7). The area under the receiver operating characteristic (ROC) curve was 0.74. The sensitivity increased in individuals with sCrAg titers of ≥1:160, CD4 count of <50 cells/mL, and disseminated central nervous system (CNS) disease (defined by a positive CSF CrAg result). However, the specificity remained relatively the same despite different sCrAg titer and CD4 count cutoffs, and disseminated disease (Table 2).

**TABLE 2:** Performance of urine and fingerprick whole blood cryptococcal antigen clinic-based testing compared to serum cryptococcal antigen laboratory-based testing^*a*^

Sample	Sensitivity % (95% CI)	Specificity % (95% CI)	Positive predictive value % (95% CI)	Negative predictive value % (95% CI)
Urine				
All (*n* = 1311)	63.8 (54.8–72.1)	84.0 (81.7–86)	29.9 (24.5–35.7)	95.6 (94.1–96.7)
sCrAg titer <1:160	55.1 (44.1–65.6)	84.0 (81.7–86)	20.5 (15.6–26.2)	96.1 (94.8–97.2)
sCrAg titer ≥1:160	84.2 (68.7–94)	84.0 (81.7–86)	14.4 (10.1–19.7)	99.4 (98.7–99.8)
CD4 count <50 cells/μL	65.3 (55–74.6)	83.5 (80.8–86)	32.3 (25.9–39.3)	95.2 (93.4–96.7)
CNS disease	91.7 (61.5–99.8)	84.0 (81.7–86.0)	5.5 (2.8–9.6)	99.9 (99.4–100)
Fingerprick whole blood				
All (*n* = 1312)	48.0 (39.1–57.1)	99.5 (98.9–99.8)	91 (81.5–96.6)	94.7 (93.3–95.9)
sCrAg titer <1:160	33.7 (24–44.5)	99.5 (98.9–99.8)	83.3 (67.2–93.6)	95.2 (93.9–96.4)
sCrAg titer ≥1:160	81.6 (65.7–92.3)	99.5 (98.9–99.8)	83.8 (68–93.8)	99.4 (98.8–99.8)
CD4 count <50 cells/μL	50.0 (39.7–60.3)	99.6 (98.9–99.9)	94.2 (84.1–98.8)	94.3 (92.5–95.8)
CNS disease	91.7 (61.5–99.8)	99.5 (98.9–99.8)	64.7 (38.3–85.8)	99.9 (99.5–100)
Fingerprick whole blood + urine				
All (*n* = 1310)	70.1 (61.2–77.7)	83.8 (81.5–85.8)	31.7 (26.3–37.5)	96.3 (94.9–97.3)

a sCrAg, serum cryptococcal antigen; CNS, central nervous system; CI, confidence interval. Fingerprick and urine results were combined for each patient and positivity was considered by 1 of the 2 methods.

***(b) Fingerprick whole blood CrAg testing. ***Of the 127 sCrAg positive samples that underwent fingerprick whole blood CrAg testing, 61 (48%) tested positive while 66 (52%) tested negative (false negative). Among the sCrAg negative patients, 0.51% (6/1,185) had false positive fingerprick whole blood CrAg LFA test results. Overall, fingerprick whole blood CrAg testing yielded a sensitivity of 48.0% (95% CI: 39.1–57.1) and a very high specificity of 99.5% (95% CI: 98.9–99.8). The PPV and NPV were 94.2% (95% CI: 84.1–98.8) and 94.3% (95% CI: 92.5–95.8), respectively. The area under the ROC curve was 0.74. Similar to urine CrAg testing, the sensitivity of the fingerprick whole blood CrAg testing increased in individuals with sCrAg titers of ≥1:160, CD4 count of <50 cells/mL, and disseminated CNS disease. However, the specificity remained relatively the same at different sCrAg titers and CD4 count cutoffs, and with disseminated disease (Table 2).

Finally, we combined urine and fingerprick results for each patient, while considering positivity by one of the two methods; a sensitivity and specificity of 70.1% (95% CI: 61.21–77.71%) and 83.77% (95% CI: 81.52–85.80%), respectively, were found. The PPV and NPV were 31.67% (26.34–37.51%) and 96.31% (94.92–97.34%), respectively.

**(ii) Agreement.** The Cohen's kappa for serum and urine CrAg testing was 0.32 (SE 0.025), indicating fair agreement between these two methods. The expected agreement was 73.7%; however, the actual observed agreement was 82.0%. For serum and fingerprick whole blood CrAg testing, the Cohen’s kappa was 0.60 (se 0.026), indicating moderate agreement between these two testing approaches. The observed agreement was 94.5% against the expected agreement of 86.2%. See Table 3.

**TABLE 3:** Cohen’s kappa statistic measure of agreement between different cryptococcal antigen testing methods^*a*^

Sample	Cohen’s kappa	Expected agreement	Observed agreement	SE	*P* value
Urine vs sCrAg	0.32	73.7%	82.0%	0.025	<0.0001
FP whole blood vs sCrAg	0.60	86.2%	94.5%	0.026	<0.0001
CSF vs urine	0.181	41.75%	52.30%	0.071	0.023
CSF vs FP whole blood	0.373	53.40%	70.77%	0.1	<0.0001

a sCrAg, serum cryptococcal antigen laboratory-based testing; FP, fingerprick; CSF, cerebrospinal fluid.

We also evaluated the agreement between CSF and urine specimen. We found a Cohen’s kappa of 0.181 (SE.0.071), indicating slight agreement between these two methods. The expected agreement was 41.75%. For CSF and fingerprick whole blood, the Cohen’s kappa was 0.373 (SE 0.1), indicating fair agreement between these two testing approaches.

## DISCUSSION

Early diagnosis and preemptive treatment for subclinical cryptococcal disease (CD) is an important strategy to prevent clinical disease and reduce mortality in patients with asymptomatic CrAg (12–15). Currently, WHO recommends the use of sCrAg LFA to screen for CD in patients with AHD before ART initiation or switch, as it was found to be highly sensitive and specific (3, 16). The CrAg LFA kit used in this study (Immuno-Mycologics Inc., Norman, OK, USA) has sensitivity and specificity of 100% when used on serum (17). This good diagnostic accuracy found by the manufacturer was based on using culture and India ink as the reference, which would likely be positive when the fungal burden is very high. Serum is routinely used in clinical care for the diagnosis of CD. However, serum is obtained through an invasive phlebotomy and the assay is typically conducted at centralized laboratories in most settings in SSA. Urine and fingerprick whole blood samples are obtained through minimally invasive means and might provide a convenient way to screen suspected cryptococcal patients.

Screening tests should have high sensitivities so as not to miss individuals who potentially have the disease who would benefit from early diagnosis and a further work-up. Although clinic-based CrAg testing on fingerprick whole blood and urine would offer advantages over laboratory-based sCrAg, we found that both lacked sufficient sensitivity to identify AHD patients with asymptomatic cryptococcal disease. Both had areas under the ROC curve of 0.74, indicating poor diagnostic performance in this patient population. When compared against the gold standard laboratory-based sCrAg testing, urine CrAg and fingerprick whole blood CrAg testing yielded a sensitivity of 63.8% and 48.0%, respectively, in screening for CD in patients with AHD with no symptoms suggestive of meningitis. However, this sensitivity was improved (70.1%) when we combined urine and fingerprick results for each patient while considering positivity by one of the two methods. Specificities were relatively higher, 84.0% for urine, 99.5% for fingerprick whole blood, and 83.7% when both urine and fingerprick results were combined. The low sensitivity (48%) using fingerprick whole blood CrAg testing in our study is comparable to Wake et al. (2018), who reported a much lower sensitivity (20%) using the same kit IMMY CrAg LFA on direct application fingerprick blood while screening for CD among patients with no symptoms or signs of meningitis (18). The authors concluded that this method may not be efficient to screen for low concentrations of CrAg among asymptomatic patients. Similar findings have been reported by Drain et al. (2019) using the same clinic-based CrAg LFA tests (IMMY Diagnostics, Norman, OK, USA) on POC clinic-based pipetted venous whole blood and direct application fingerprick whole blood among newly-diagnosed HIV-infected adults in South Africa, though not entirely asymptomatic as some participants described having symptoms commonly associated with cryptococcal meningitis (19). The authors found a decreased diagnostic accuracy of the CrAg LFA (venous whole blood 46% [95% CI 19–75%] and fingerprick whole blood 38% [95% CI 14–68%] compared to the enzyme immunoassay (EIA) used as the gold standard).

Our study finding deviates from the manufacturer’s kit insert, which reported a sensitivity of 99.3% (95% CI 96.3–99.9) and specificity of 94.4% (95% CI 90.2–97.2) when whole blood was used. This difference may be largely due to the fact that the manufacturer was using culture and India ink positive samples as the reference; this would imply that the samples were from individuals with a higher fungal burden and likely cryptococcal disease, whereas our study population was asymptomatic. However, an important limitation raised by the manufacturer is that finger stick whole blood should be measured with a pipette for proper accuracy (8); in our implementation study we used direct application of a fingerprick whole blood droplet or immersion of the device into urine and hence cannot ascertain the recommended volume of blood was used for either urine or fingerprick whole blood POC tests. Wake et al. (2018) also found that pipetting the correct volume of fingerprick blood improved diagnostic performance over direct application of fingerprick blood to the CrAg LFA sample pad (18). However, our approach was more consistent with the approach that would be anticipated for a clinic-based POC assay.

In our study, urine CrAg and fingerprick whole blood CrAg testing yielded a sensitivity of 63.8% and 48.0%, respectively, in screening for CD in patients with no symptoms suggestive of meningitis. The accuracy of the IMMY CrAg LFA was also assessed among patients with no symptoms or signs of meningitis in South Africa in a validation study. Sensitivities of 82% and 100% were found after direct application of the device to fingerprick blood and using a pipette to transfer blood, respectively (18). Previous studies have reported high sensitivity and specificity of fingerprick whole blood CrAg screening with reports of 100% agreement between fingerprick whole blood, and serum and plasma CrAg LFA (20, 21). However, these studies were conducted among patients with CD who likely had a higher fungal burden and hence higher CrAg titers.

Although we found relatively low sensitivities of CrAg in fingerprick and urine among asymptomatic individuals presenting for HIV primary care with CD4 counts below 200 cells/μL, the sensitivities of these 2 screening approaches increased at higher sCrAg titers (≥1:160), at lower CD4 count (<50 cells/μL), and with disseminated asymptomatic CNS disease (all 3 being risk factors or proxies of increased fungal burden). Our study agrees with that of Wake et al. (2018), who showed that sensitivity improved when testing asymptomatic patients with higher CrAg titers (18). Our findings also support previous studies (21–24) that reported good diagnostic performance of both urine and fingerprick whole blood CrAg screening in symptomatic patients (and thus higher crAg titers). We also found that combining urine and fingerprick results for each patient, while considering positivity by one of the two methods, improved the sensitivity. This testing algorithm could improve the screening of CD among AHD patients with asymptomatic cryptococcal disease in resource-limited settings given the fact that both tests can be implemented in a clinic-based POC setting. However, more studies are required to evaluate the diagnostic performance of this testing approach.

### Study strengths.

Our study had several strengths. For instance, we were able to implement POC screening of urine and fingerprick CrAg on a large scale with over 1,000 AHD patients enrolled, which is important for the translation of such studies into clinical practice. The large sample size increased the power and precision of this study and enabled validation of smaller studies that had similar findings. We were able to detect asymptomatic cryptococcal antigenemia (an independent predictor of meningitis and death [14, 25]) among individuals with AHD, in line with literature that reports a prevalence of 1%–15% (26, 27) in this population group; however, our results show that both urine and fingerprick CrAg, as implemented in our study, are a poor alternative to laboratory-based screening. The higher sensitivities observed with increasing serum CrAg titers suggests that urine and fingerprick CrAg could potentially be useful in symptomatic individuals as a POC test in a resource-limited setting with no laboratory facilities. The specificity of urine found in our study could be improved by preheating the urine for a short period of time prior to CrAg testing, as reported by Brito-Santos et al. (2017) (22).

Our study was designed such that POC screening preceded lab testing. Furthermore, laboratory technicians were blinded to the POC test results, and the final sCrAg results were read by 3 different laboratory technicians independently; the double-blinding strategy minimized reporter bias. Furthermore, the kit used in this study, CrAg LFA kit (IMMY, Norman, OK), has an extremely high diagnostic performance, allowing the qualitative and quantitative determination of antigenemia in serum (our reference/gold standard in this study) and CSF in individuals with culture and India ink positive CD. Taken together, these strengths increased the level of certainty and confidence in our study results.

### Study limitations.

Our study had several limitations. Firstly, because results are interpreted visually, the ability to discern the presence/absence of a test line is critical; a high background color from fingerprick blood could potentially obscure the ability to visualize faint positive lines, leading to false negative fingerprick whole blood CrAg interpretations.

An important limitation was the inability to ascertain the volume of sample assayed in the clinic-based POC settings following the techniques used in our study. The volume of fingerprick blood applied to the CrAg device could have been inadequate, below the limit of detection, thereby reducing the probability of the reader discerning the presence of a test line; this would be a problem in samples from participants with low fungal burdens and could account for some of the false negatives observed in our study. As alluded to before, pipetting the correct volume of fingerprick whole blood improved the sensitivity of fingerprick CrAg LFA elsewhere (18).

Another limitation in our study was that we did not quantify CrAg titers in urine and fingerprick blood independently e.g., via glucuronoxylomannan (GXM) Elisa. The IMMY CrAg LFA detects glucuronoxylomannan (GXM), and independently quantifying GXM in the samples would have provided further delineation to help clarify whether discordances were due to technical aspects of POC urine and fingerprick blood testing or due to different CrAg titers in the respective sample types (28).

False negative results might have also arisen due to the hook (prozone) effect, which is observed at extremely high titers (above antigen concentrations of >0.14 mg/L). This results in faint positive or even false negative results due to excess antigen binding colloidal gold in preference to immobilized antibody in the test area of the strip. However, this is unlikely in this study, where the participants were asymptomatic and most likely had low fungal burden (median sCrAg titer: 1:20, IQR 1:5–1:160).

There was also a risk of obtaining false positive results due to cross-reactivity between cryptococcal and aspergillus as per the same kit. Individuals with AHD often suffer from multiple coinfections, which could have also interfered with the results.

### Conclusion.

Clinic-based urine and fingerprick whole blood CrAg testing as POC tests performed better in screening for CD among AHD patients with CNS disease than in diagnosing subclinical CD in asymptomatic AHD patients without CNS involvement. Strategies to improve the sensitivity of urine and fingerprick clinic-based POC CrAg tests could enable testing of urine and fingerprick to play a potential role in improving early diagnosis of CD among asymptomatic patients with AHD in many resource-limited settings.

## MATERIALS AND METHODS

### Study design.

This was a cross-sectional study where we evaluated the diagnostic accuracy of CrAg LFA on fingerprick whole blood and urine samples collected from individuals presenting to care with CD4 of <200 cells/μL. Participants were recruited as part of a longitudinal study (the CryptoART study) (11) and samples collected at enrollment were used for this substudy. Briefly, the CryptoART study was an implementation of a “screen and treat” program for subclinical CD using an LFA (Immuno-Mycologics Inc., Norman, OK, USA) in adults (18 years and above) with AHD before ART initiation or switch from a failing ART regimen. Consented patients underwent the study procedures, which included collecting samples such as fingerprick whole blood and urine for clinic-based POC and venous blood for laboratory-based testing.

### Inclusion criteria.

Adult participants (age ≥18 years) with AHD (CD4 count of <200 cells/μL) residing within 50 km of Harare and able to provide written informed consent were enrolled.

### Exclusion criteria.

In the CryptoART study, participants were excluded if they presented with clinical symptoms suggestive of meningitis or had a recent history of CM within 2 weeks of enrollment. Individuals underwent laboratory screening tests for eligibility, and those with severe hepatic injury, jaundice, alanine transferase (ALT) >5× the upper limit of normal, and with renal failure, defined by an estimated Glomerular Filtration Rate (eGFR) of ≤30mL/min (using the MDRD [Modification of Diet in Renal Disease] equation), were also excluded from the study. Any woman who was found to be pregnant at the time of eligibility screening (detected by the urine pregnancy test) was not eligible to participate in the study, and individuals who had had a previous allergy or other reaction to amphotericin B and/or fluconazole were also excluded from the study (11).

### Study setting.

The CryptoART study was conducted at 20 outpatient community-based clinics in Harare, Zimbabwe. The lateral flow assays on urine and fingerprick whole blood were conducted at the point of care in the recruiting clinics.

### Study population.

A total of 1,333 HIV-positive individuals, aged ≥18 years with CD4 counts <200 cells/μL, but with no signs and symptoms suggestive of meningitis, were enrolled from 20 outpatient clinics in Harare. These participants were then followed up for a maximum of 12 months from the day of enrollment into the study. Blood and urine samples (and CSF where indicated) were collected at enrollment (day 1) and on other subsequent visits as per the study protocol. Of the 1,333 participants enrolled, 132 (9.9%) were serum CrAg (sCrAg) positive.

### Selection of participants for this study.

All participants who were successfully enrolled into the CryptoART study were eligible for inclusion into this study. Samples included in this analysis are those from CryptoART study participants who were able to provide urine samples at the time of enrollment, and from patients for whom POC fingerprick whole blood testing was done when kits were available at the clinic.

### Study procedures.

Patients were screened for study enrollment eligibility, and those who passed the screening process were enrolled into the study before undergoing the study procedures, which included collecting samples such as fingerprick whole blood and urine for clinic-based POC testing, and venous blood for laboratory-based testing. Venous blood, fingerprick blood, and urine were collected according to the CryptoART study sample collection standard operating procedures by trained research nurses at the clinic. The research nurses participated in an in-house quality assurance program that included blinded retesting and direct observation *in situ*. CrAg testing was performed using the CrAg LFA test (IMMY Inc, Norman Oklahoma) (17). Venous blood was sent to the lab for sCrAg testing, while fingerprick and urine CrAg testing was conducted at the POC by the trained research nurses.

### Whole blood fingerprick CrAg LFA.

Positive and negative (diluent) kit controls were run successfully daily before testing any patient samples. The expiry dates and storage conditions of the kits were monitored and documented; expired kits were not used. The fingerprick whole blood sample was collected by pricking the pad of the index finger with a lancet following sterilization of the finger with an alcohol swab. The first droplet of blood was wiped off and the next free-flowing droplet was applied directly onto the sample pad of a CrAg LFA test device. The device was immediately immersed into a drop of sample diluent in a tube and removed 10 min later for interpretation by the research nurse.

### Urine CrAg LFA.

Ten mL of midstream urine was collected into a BD urine collection jar by the participant. Urine CrAg testing was performed immediately on receipt of the urine by the research nurse by submersing the sample pad of a CrAg LFA device into the urine for 1–2 s, and then immersing the device into a tube with a drop of sample diluent. Finally, the CrAg LFA device was removed 10 min later for interpretation by a research nurse.

### Serum CrAg LFA.

Whole blood was collected into a plain tube (no anticoagulant BD tube). The tube was placed on a tube rack in a cooler box with ice pack and transported to the lab within 2 h of collection. The whole blood was centrifuged at 1,714 × *g* for 5 min, and serum was harvested. sCrAg testing was performed as per kit insert; briefly, 40 μL of serum was pipetted into a tube containing a drop (40 μL according to the kit insert) of sample diluent, and the sample pad of the CrAg device was immersed into the mixture and incubated for 10 min then removed for independent readings by 3 laboratory scientists. The CrAg LFA in serum samples was considered the reference standard (17). sCrAg titers were determined for sCrAg positive samples as per manufacturer’s instructions. sCrAg positive participants who consented to an LP had their CSF tested using the same procedure described above for sCrAg testing. . Briefly, 40 μL of CSF was pipetted into a tube with a drop of sample diluent, and the sample pad of the CrAg device was immersed into the mixture and allowed to incubate for 10 min then removed for interpretation. CSF CrAg titers were also determined in positive specimens. The laboratory scientists who performed these tests were generally blinded to the POC test results obtained by the research nurses at the study sites.

### Statistical analysis.

Data were analyzed using Stata version 17.0 (StataCorp, College Station, TX, USA). Baseline demographic and clinical characteristics were summarized in a table using medians and interquartile ranges (IQR) and absolute numbers and proportions for categorical variables. Wilcoxon rank sum tests were applied to compare sCrAg group medians for continuous variables and Chi-squared tests for frequency distribution of categorical characteristics between sCrAg groups. CrAg titers among sCrAg positive participants were summarized using medians and IQRs.

We compared the performance of the clinic-based fingerprick whole blood and urine CrAg LFA screening methods against the reference/gold standard laboratory-based sCrAg screening method to determine the sensitivity, specificity, positive predictive values (PPVs), and negative predictive values (NPVs) at 95% confidence interval (CI) by a diagnostic algorithm in Stata. The Cohen’s kappa was used to determine the level of agreement between the gold standard sCrAg and the fingerprick whole blood and urine CrAg test results. The Cohen’s kappa takes into account the observed agreement and the expected agreement in the formula (k = P_0_–P_e_/1–P_e_), where k represents the Cohen’s kappa, P_0_ is the relative observed agreement, and Pe is the expected agreement. This expected agreement was determined as the overall probability that both the gold standard sCrAg and the fingerprick whole blood and urine CrAg test occur by chance. The strength of the kappa coefficient was interpreted as follows: ≤0, no agreement; 0.01–0.20, slight agreement; 021–0.40, fair agreement; 0.41–0.60, moderate agreement; 0.61–0.80, substantial agreement; and 0.81–1.00, strong or almost perfect agreement (29).

### Ethics approvals.

The CryptoART study was approved by the Joint Research Ethics Committee (JREC) for the University of Zimbabwe College of Health Sciences and the Parirenyatwa Groups of Hospitals (JREC/01/13), Medical Research Council of Zimbabwe (MRCZ) (MRCZ/A1767), Research Council of Zimbabwe (RCZ), Partners Human Research at Massachusetts General Hospital, and the Centers for Disease Control and Prevention (CDC) institutional review board. All participants with samples used in this study had provided a written informed consent for sample storage, future use. and shipment at the time of enrollment into the parent CryptoART study.
